# Supplementary material for: Drug repurposing for aging research using model organisms
Source: Aging Cell. 2017 Jun 16;16(5):1006–15. doi: 10.1111/acel.12626 (PMC5595691; doi:10.1111/acel.12626)
Supplement: Supplementary file 7 — Data S1 Zip‐Archive of all report cards. [file ACEL-16-1006-s007.zip › RC_PYR.pdf]

PYR

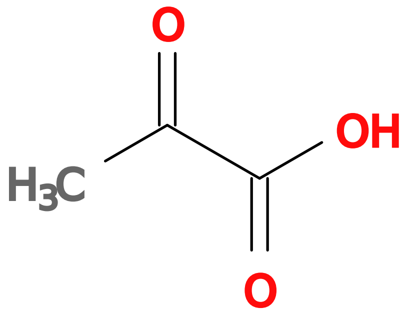

Database identifiers

ChEMBLCompound	CHEMBL1162144
DrugBank	DB00119
CHEBI	32816
eMolecules	478132

Ranking

	Rank	Score
Drosophila	516/697	0.25
C. elegans	294/591	0.195

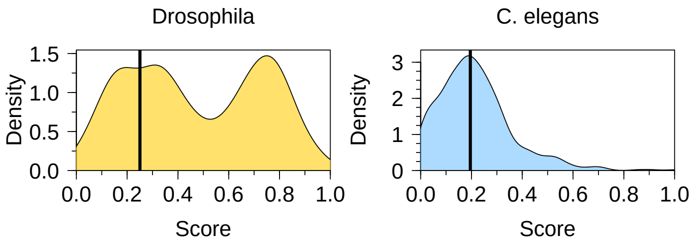

	Ageing implication	Domain conservation	Binding site conservation	Binding affinity	Bioavailability	Lipinski	Promiscuity	Purchasability	Drug approval	**Total**
Drosophila	0.36	0.94	1.0	0.249	(0.9)	0.0	-0.0	0.1	0.075	0.25
C. elegans	0.36	0.935	1.0	0.249	0.25	0.0	-0.0	0.1	0.075	0.195

Names

- **pyruvic acid**
- 2-ketopropionic acid
- 2-Oxopropanoate
- 2-oxopropanoic acid
- 2-Oxopropionsaeure
- acetylformic acid
- acide pyruvique
- alpha-ketopropionic acid
- alpha-Oxopropionsaeure
- Brenztraubensaeure
- BTS
- Ch3cocooh

· Pyroracemic acid

Roles

fundamental metabolite

Status

Approved drug (according to ChEMBL)	No
Classification (according to DrugBank)	approved, nutraceutical
Number of Rule of 5 violations	0
Binding affinity to original target in log units (RF-Score prediction)	3.9
Burns *C. elegans* bioavailability prediction	-2.08

Medical Information

Indicatation: For nutritional supplementation, also for treating dietary shortage or imbalance
Mechanism of action: Pyruvate serves as a biological fuel by being converted to acetyl coenzyme A, which enters the tricarboxylic acid or Krebs cycle where it is metabolized to produce ATP aerobically. Energy can also be obtained anaerobically from pyruvate via its conversion to lactate. Pyruvate injections or perfusions increase contractile function of hearts when metabolizing glucose or fatty acids. This inotropic effect is striking in hearts stunned by ischemia/reperfusion. The inotropic effect of pyruvate requires intracoronary infusion. Among possible mechanisms for this effect are increased generation of ATP and an increase in ATP phosphorylation potential. Another is activation of pyruvate dehydrogenase, promoting its own oxidation by inhibiting pyruvate dehydrogenase kinase. Pyruvate dehydrogenase is inactivated in ischemia myocardium. Yet another is reduction of cytosolic inorganic phosphate concentration. Pyruvate, as an antioxidant, is known to scavenge such reactive oxygen species as hydrogen peroxide and lipid peroxides. Indirectly, supraphysiological levels of pyruvate may increase cellular reduced glutathione.
Toxicity: Those taking large doses of supplemental pyruvateamp;mdash;usually greater than 5 grams dailyamp;mdash;have reported gastrointestinal symptoms, including abdominal discomfort and bloating, gas and diarrhea. One child receiving pyruvate intravenously for restrictive cardiomyopathy died.
Metabolism: In the liver, pyruvate is metabolized via several pathways.

(Information from DrugBank)

Compound Target Characteristics

Phosphoenolpyruvate carboxykinase, cytosolic [GTP]

Best gene implication in ageing for this target family came from gene Q9Z2V4 via mapping the annotation from Ensembl ENSMUSG00000027513 via mapping the annotation from EntrezGene 18534 via mapping the annotation from GenAgeModels 0497 annotated in GenAge release 17.

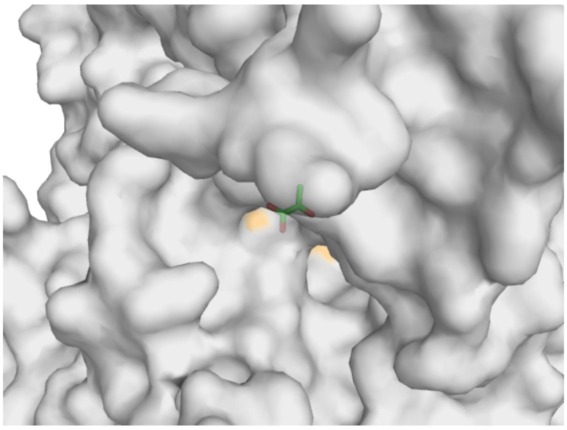

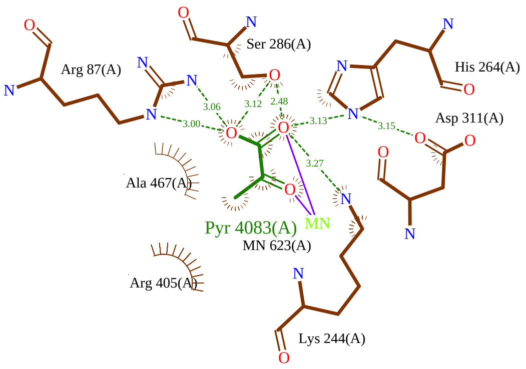

protein	amino acids contacts (binding site)
PDB:2qf2:chainA:P07379	R K H S D R A
sp:P35558:PCKGC_HUMAN	R K H S D R A
sp:P07379:PCKGC_RAT	R K H S D R A
sp:Q9Z2V4:PCKGC_MOUSE	R K H S D R A
tr:A8DYI3:A8DYI3_DROME	R K H S D R A
tr:Q7JXB5:Q7JXB5_DROME	R K H S D R A
sp:P20007:PCKG_DROME	R K H S D R A
tr:Q7JKI1:Q7JKI1_CAEEL	R K H S D R A
tr:Q7JKI3:Q7JKI3_CAEEL	R K H S D R A
tr:Q8ITY0:Q8ITY0_CAEEL	R K H S D R A
tr:Q7JKI2:Q7JKI2_CAEEL	R K H S D R A
tr:O44906:O44906_CAEEL	R K H S D R A
tr:O02286:O02286_CAEEL	R K H S D R A

	whole protein		domain-based		contact-based	
protein	ident	simil	ident	simil	ident	simil
PDB:2qf2:chainA:P07379	1.0	1.0	1.0	1.0	1.0	1.0
sp:P35558:PCKGC_HUMAN	0.91	0.98	0.9	0.97	1.0	1.0
sp:P07379:PCKGC_RAT	1.0	1.0	1.0	1.0	1.0	1.0
sp:Q9Z2V4:PCKGC_MOUSE	0.98	0.99	0.97	0.99	1.0	1.0
tr:A8DYI3:A8DYI3_DROME	0.6	0.8	0.62	0.82	1.0	1.0
tr:Q7JXB5:Q7JXB5_DROME	0.62	0.85	0.65	0.88	1.0	1.0
sp:P20007:PCKG_DROME	0.61	0.84	0.64	0.87	1.0	1.0
tr:Q7JKI1:Q7JKI1_CAEEL	0.47	0.72	0.49	0.75	1.0	1.0
tr:Q7JKI3:Q7JKI3_CAEEL	0.55	0.82	0.57	0.84	1.0	1.0
tr:Q8ITY0:Q8ITY0_CAEEL	0.5	0.73	0.54	0.78	1.0	1.0
tr:Q7JKI2:Q7JKI2_CAEEL	0.56	0.84	0.58	0.87	1.0	1.0
tr:O44906:O44906_CAEEL	0.54	0.81	0.59	0.86	1.0	1.0
tr:O02286:O02286_CAEEL	0.54	0.82	0.58	0.87	1.0	1.0

Pepck (UniProt:P20007) annotation

Function: Catalyzes the conversion of oxaloacetate (OAA) to phosphoenolpyruvate (PEP), the rate-limiting step in the metabolic pathway that produces glucose from lactate and other precursors derived from the citric acid cycle.

Cofactor: Mn(2+)Note=Binds 1 Mn(2+) ion per subunit. ;

Pathway: Carbohydrate biosynthesis; gluconeogenesis.

Subunit: Monomer.

(Information from UniProt)
